# Design of a Soft Robotic Elbow Sleeve with Passive and Intent-Controlled Actuation

**DOI:** 10.3389/fnins.2017.00597

**Published:** 2017-10-25

**Authors:** Tze Hui Koh, Nicholas Cheng, Hong Kai Yap, Chen-Hua Yeow

**Affiliations:** ^1^Evolution Innovation Laboratory, Department of Biomedical Engineering, National University of Singapore, Singapore, Singapore; ^2^NUS Graduate School for Integrative Sciences and Engineering, National University of Singapore, Singapore, Singapore

**Keywords:** robotic, rehabilitation, elbow, stroke, assistive, electromyography-driven, wearable, soft-robotic

## Abstract

The provision of continuous passive, and intent-based assisted movements for neuromuscular training can be incorporated into a robotic elbow sleeve. The objective of this study is to propose the design and test the functionality of a soft robotic elbow sleeve in assisting flexion and extension of the elbow, both passively and using intent-based motion reinforcement. First, the elbow sleeve was developed, using elastomeric and fabric-based pneumatic actuators, which are soft and lightweight, in order to address issues of non-portability and poor alignment with joints that conventional robotic rehabilitation devices are faced with. Second, the control system was developed to allow for: (i) continuous passive actuation, in which the actuators will be activated in cycles, alternating between flexion and extension; and (ii) an intent-based actuation, in which user intent is detected by surface electromyography (sEMG) sensors attached to the biceps and triceps, and passed through a logic sequence to allow for flexion or extension of the elbow. Using this setup, the elbow sleeve was tested on six healthy subjects to assess the functionality of the device, in terms of the range of motion afforded by the device while in the continuous passive actuation. The results showed that the elbow sleeve is capable of achieving approximately 50% of the full range of motion of the elbow joint among all subjects. Next, further experiments were conducted to test the efficacy of the intent-based actuation on these healthy subjects. The results showed that all subjects were capable of achieving electromyography (EMG) control of the elbow sleeve. These preliminary results show that the elbow sleeve is capable of carrying out continuous passive and intent-based assisted movements. Further investigation of the clinical implementation of the elbow sleeve for the neuromuscular training of neurologically-impaired persons, such as stroke survivors, is needed.

## 1. Introduction

According to the World Health Organization, every year approximately 15 million people around the world suffer from stroke (Rodrigo et al., 2013), and 70–80% experience upper-extremity impairment (Hu et al., 2007). The upper extremity, which includes the shoulder, elbow, wrist and hand, is important for the spatial movement and manipulation of objects, as well as for gesturing. It is therefore essential that stroke survivors regain a certain level of functionality so that they are able to independently carry out the activities of daily living.

Traditionally, stroke rehabilitation is done in the form of therapeutic intervention with the help of physiotherapists and occupational therapists, which, while effective, is time-consuming and labor intensive (Hu et al., 2007). To reduce the burden on caregivers and the need for oversight by trained therapists, robotic devices have been developed, which aim at supporting the patients in carrying out rehabilitation at their convenience and with minimal supervision. These robotic devices are typically devised to carry out: (1) continuous passive movements, and (2) active-assisted movements. Continuous passive movements are useful for reducing muscle tone to improve the mobility of the joint (Hu et al., 2009), while active-assisted movements help in recovering motor control through the positive shaping of the cortical reorganization following brain injuries such as stroke (Hesse et al., 2003). Current robotic rehabilitation devices include end-effector robots, such as the MIT Manus and MIME, and exoskeletons, such as the Myomo, ARMin, OrthoJacket and KIST Wearable Robotic Arm (Zhang et al., 2015). While these devices have demonstrated good efficacy in rehabilitation, they are costly, non-portable and rigid, which limits them to the clinical setting, and their rigidity also makes it difficult for them to be aligned with the patients' joints during rehabilitation (Oguntosin et al., 2015).

Soft robotics is an emerging area of robotics research resulting from the melding of materials chemistry and robotics, and aims to tackle the challenges faced by traditional robotics (Polygerinos et al., 2015b). As its name suggests, soft robotics consists of robots that are manufactured with flexible or elastomeric structural elements. In particular, soft pneumatic actuators are an important component used in soft wearable robots because they are lightweight, flexible and able to distribute pressure evenly along the joint (Oguntosin et al., 2015; Polygerinos et al., 2015a,b). Additionally, their response to actuation may be customized to a large degree to accomplish motions typically difficult with hard robotic devices, possibly overcoming alignment problems through the direct attachment of actuators to the limbs (In et al., 2015). Some current developments in soft robotics for rehabilitative purposes include a soft robotic glove for robot-assisted hand therapy (Yap et al., 2015, 2016b; Yeo et al., 2016), as well as a soft robotic sock for robot-assisted ankle exercise (Low et al., 2016).

The provision of timely assistance upon detecting user-intent (Lenzi et al., 2012) is a common issue in the development of robotic devices for active-assisted rehabilitation. Current state-of-the-art devices such as the Myomo and OrthoJacket have built-in electromyography (EMG) capabilities that help to estimate the joint torque needed to perform movement (Benitez et al., 2013). Studies have shown that intention-based rehabilitation enhances the therapeutic effect, accelerating the speed with which functionality is recovered (Zhang and Zhou, 2012).

The above-mentioned developments open up possibilities in developing myoelectric-based lightweight wearable robotics, which have the potential to both restore normal function in patients affected by stroke, as well as to enhance the outcome of their neuro-muscular rehabilitation. In this paper, we present the design of a soft robotic elbow sleeve for enabling flexion and extension of the elbow both passively and intent-based motion reinforcement, which may be useful as a neuro-muscular training device. In the following sections, we discuss the methods for fabricating the actuators for the elbow sleeve, the design of the control system for the elbow sleeve, methods for mechanical characterization of the actuators, as well as a description of the range of motion study that was conducted to evaluate the functionality of the device. Finally, we present the results of the mechanical characterization and the range of motion study, and discuss their implications.

## 2. Materials and methods

### 2.1. Design overview

**Figure 1:**
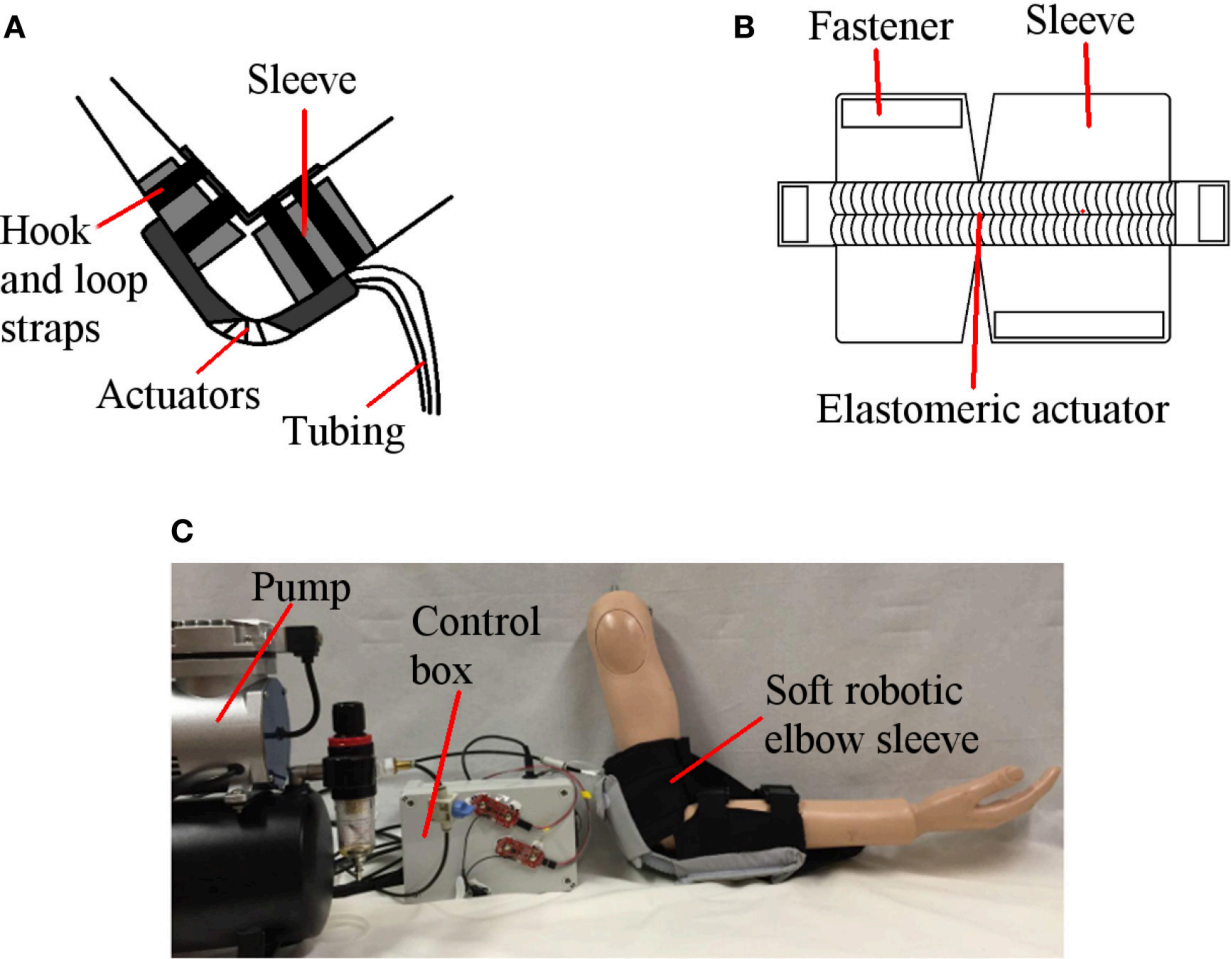
Soft robotic elbow sleeve for stroke rehabilitation. **(A)** Illustration of the soft robotic sleeve. **(B)** Schematic of the constraining layer for the flexion actuator. **(C)** Fully assembled system.

The soft robotic elbow sleeve spans across the elbow joint, from below the biceps of the arm to the middle of the forearm (Figure 1), and comprises the following components: (1) two pneumatic actuators to individually effect elbow joint flexion and extension; (2) a programmable pump-valve control system to switch between modes of operation; (3) surface EMG sensors which are attached to the biceps and triceps; (4) an external pump for providing air supply to the actuators; and (5) hook and loop straps which secure the actuators to the arm.

**Figure 2:**
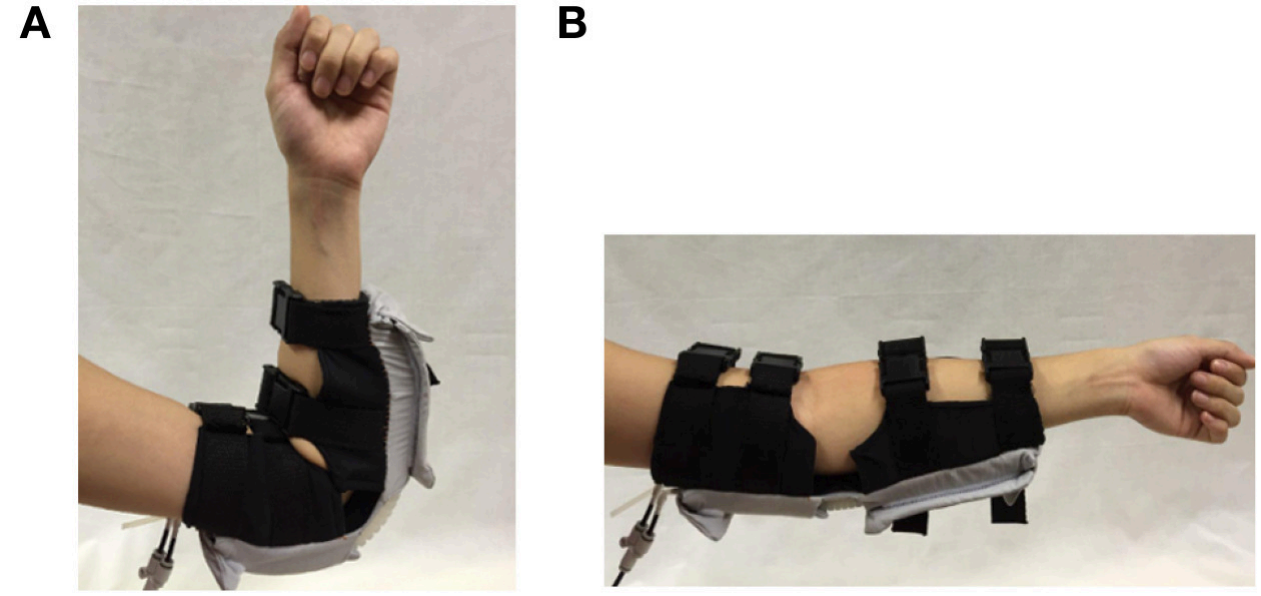
Arm configurations during the use of the soft robotic elbow sleeve. **(A)** Flexed configuration. **(B)** Extended configuration.

The working principle of the soft robotic elbow sleeve is based on the controlled air pressurization of two separate pneumatic actuators working antagonistically with respect to one another. Mechanically, pressurization of the flexion actuator causes the elbow to flex, while pressurization of the extension actuator causes the elbow to extend. The two resulting configurations (flexed and extended) of the arm are shown in Figure 2. Electronic control for the mechanical action of the actuators allows for two distinct mode of operations of the soft robotic sleeve—passive rehabilitation and intent-based rehabilitation. This is accomplished through the valves, pressure sensor, micro-controller, which are packed within a custom-made control box, and two surface EMG sensors. The details of the actuators and the control system are given in the following sections.

### 2.2. Actuator design and fabrication

#### 2.2.1. Flexion actuator

**Figure 3:**
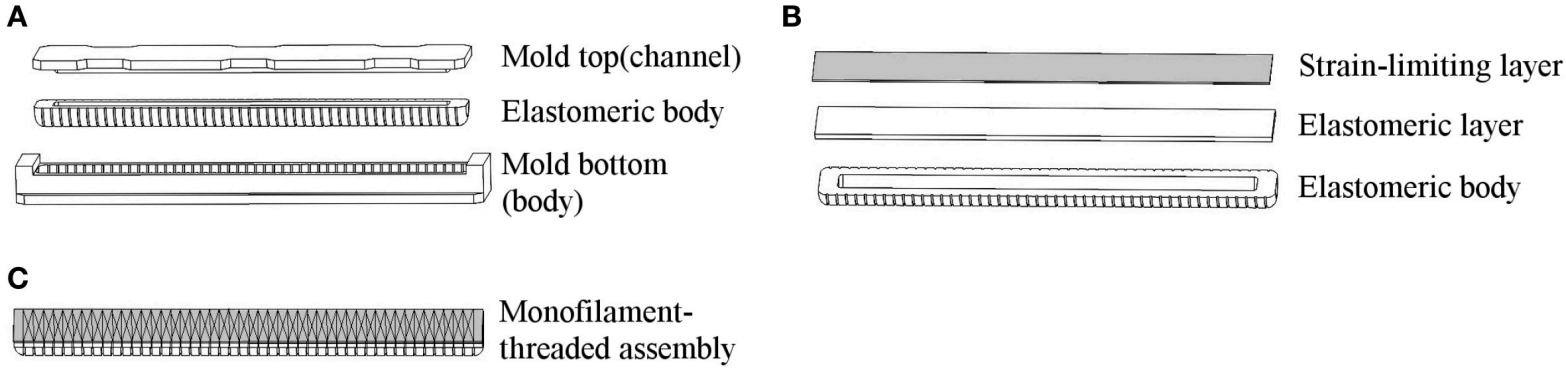
Key stages of actuator fabrication. **(A)** Molding of the actuator's elastomeric body (Dragon Skin 20) using a 3D-printed mold (ABS). **(B)** Application of a thin elastomeric layer (Dragon skin 20) together with strain limiting layer (Nylon). **(C)** Winding of a monofilament thread (Nylon) in a double-helical pattern to provide radial reinforcement.

**Figure 4:**
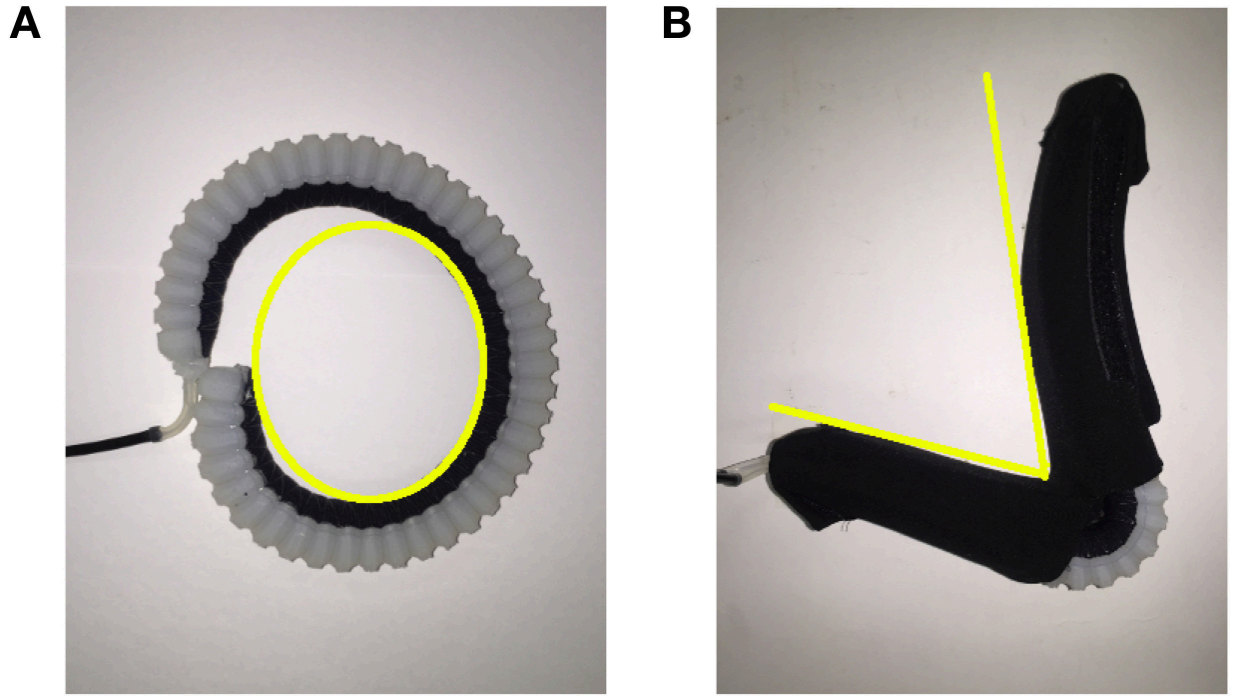
Illustrating the effect of wrapping Nylon around the flexion actuator to acquire a desired angular configuration. **(A)** An unconstrained elastomeric actuator in flexed formation, with the yellow curve indicating a rounded curvature upon air pressure input. **(B)** A fabric constrained elastomeric actuator upon pressurization, with the yellow lines indicating the angular curvature achieved.

The flexion actuator in the soft robotic sleeve is an elastomeric construct that bends upon an input air pressure. To obtain a desired angular configuration upon an input air pressure, a fiber-fabric-reinforced approach adapted from Polygerinos et al. (2015b) was adopted in the development of the flexion actuator. The molds for the flexion actuator body (which described a length of 24 cm and radius of 1 cm) were first designed using a Computer-Aided Design software (Dassault Systèmes SOLIDWORKS Corp., USA) and 3D-printed with Acrylonitrile Butadiene Styrene (ABS). These molds were then used to fabricate the actuator body using a soft elastomeric material, Dragon Skin 20 (Figure 3A). The actuator body was then sealed with a flat piece of elastomeric layer (Dragon Skin 20), and a woven nylon strip (which acts as a strain-limiting layer) was glued to this flat surface to enable bending of the actuator (Figure 3B). Following that, monofilament thread (Nylon) was hand-wound around the actuator, with the thread pitch (5 mm) determined by the grooves imprinted onto the actuator (Figure 3C). This serves to radially reinforce the actuator. Two of these flexion actuators were made and assembled for the elbow sleeve. They were wrapped with a Nylon textile where bending is not required, so as to allow for an angular configuration in bending (Figure 4).

Dragon Skin 20 was chosen as the elastomer for fabricating the actuator due to its ability to withstand higher pressure and provide higher force output at Shore A hardness of 20 as compared to other tested materials such as Dragon Skin 10. The length of the actuator (24 cm) was determined based on the distance of the center of gravity of the average human forearm from the elbow joint (approximately 41.7% from the elbow joint, which is 18.7 cm, inclusive of hand) (Drillis et al., 1964) as well as the force that each actuator is able to produce, in order to ensure that sufficient torque can be generated to assist flexion. The Nylon monofilament thread that was wound around the actuator was introduced to limit radial expansion and thus improve the actuator's durability.

#### 2.2.2. Extension actuator

As the extension actuator is placed interior to the flexion actuator along the elbow sleeve, we cannot simply replicate the method used for the flexion actuator (elastomeric bending actuator) in developing the extension actuator as it would hinder the operation of the flexion actuator due to its sheer volume. An alternate design, following an inflatable beam model, was instead utilized, involving the use of the torque generated from the inflation of the actuator to extend the elbow.

**Figure 5:**
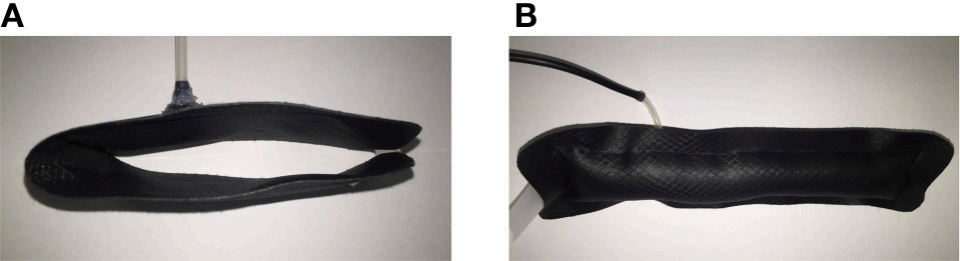
Illustrating the two configurations of the extension actuator, with and without air pressurization. **(A)** The deflated nylon ripstop actuator in flexed configuration, which allows conformation to the flexion actuator. **(B)** The inflated nylon ripstop actuator in extended configuration, which acts as a rigid beam and resists bending deformation.

Nylon ripstop sheets coated with thermoplastic polyurethane were heat sealed to form an air tight chamber with a single inlet, resulting in a fabric actuator with a length of 20 cm. While deflated, the actuator does not take up much space, and provides little hindrance to the action of the flexion actuator (Figure 5A). Through inflation, the actuator is then able to act in a manner similar to that of a rigid beam and resist deflection (Sanan et al., 2009; Yap et al., 2016c) (Figure 5B).

### 2.3. Actuator characterization

**Figure 6:**
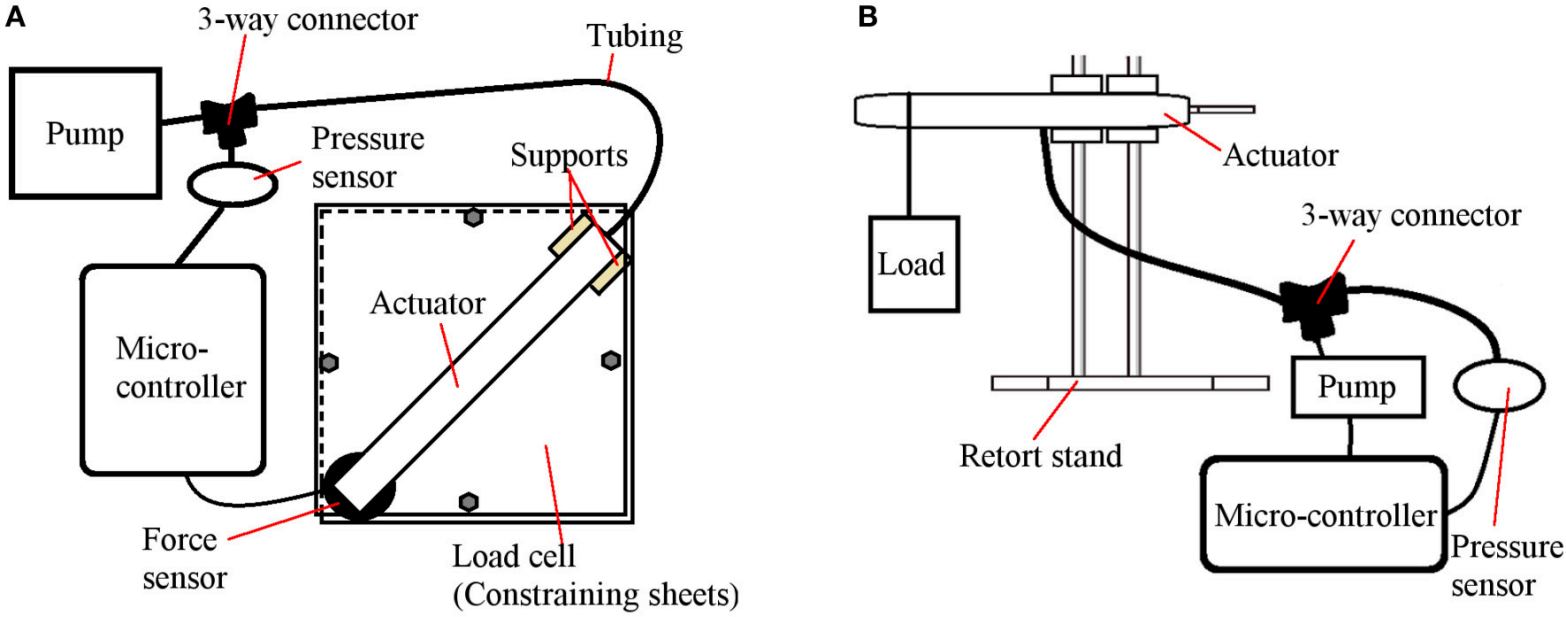
Experimental platforms for the characterization of the actuators. **(A)** Flexion actuator experimental platform. **(B)** Extension actuator experimental platform.

To evaluate the force-pressure relationship of the flexion and extension actuators, two experimental platforms were developed, as illustrated in Figure 6.

For the flexion actuator (Figure 6A), following a similar protocol introduced by Polygerinos et al. (Polygerinos et al., 2015b; Yap et al., 2016a), the actuator was constrained in a rigid fixture to minimize nonlinear effects, with the distal tip placed in contact with a force sensor. The pressure in the actuator was gradually increased as the force sensor recorded the bending force exerted by the tip. This enables the measurement of the maximum force generated by the tip of the actuator at each pressure.

For the extension actuator (Figure 6B), experimental deflection tests were conducted by fixing the actuator at one end with varying weights hung from the other end of the actuator. For each weight, the pressure was decreased to the point at which the actuator began to deflect. This setup is suitable for determining the relationship between pressure and load capacity. A similar mode of testing was utilized by Sanan et.al to find the relationship between load and deflection (Sanan et al., 2009).

### 2.4. Control system

The programmable pump-valve control system of the robotic elbow sleeve allows for switching between the two rehabilitation modes—passive and active modes—and uses a commercial pneumatic pump and valve for the supplying of air pressure for actuation, controlled by an Arduino Uno micro-controller. Two pneumatic pressure sensors are used to individually monitor the pressures within the flexion and extension actuators, and two surface EMG sensors are used for intent-detection by measuring EMG signals from the biceps and triceps. Feedback control of the system for both passive and EMG rehabilitation modes uses a closed-loop proportional-integral-derivative (PID) regulator, following the basic equation:

(1) $$\begin{array}{r} u\left(t\right)=K_{p}e\left(t\right)+K_{i}\int_{0}^{t}e\left(t\right)\text{d}t+K_{d}\frac{\text{d}e\left(t\right)}{\text{d}t} \end{array}$$

where the *u*(*t*) is the control variable, in this case, the pressure value; *e*(*t*) is the error value; *K*_*p*_, *K*_*i*_, and *K*_*d*_ are the proportional, integral, and derivative gains, respectively; and *t* is time. The controller uses pressure as an input and in turn regulates the duty cycle of the pulse-width-modulation (PWM) as a means to control the pressure according to a pre-set point. This also acts as a safety feature to ensure that the pressure within the actuators do not reach failure point. To streamline the process of switching between modes, calibrating the EMG sensors, and the processing of generated data, a Graphical User Interface (GUI) was specially developed in C++ using the Processing Integrated Development Environment.

In the passive rehabilitation mode, the device is actuated in cycles, alternating between flexion and extension. This continuous repetitive movement was enabled by utilizing the internal timer of the micro-controller to track time. When the defined time interval was reached, the system would switch from flexion to extension and vice versa, and the timer would be reset.

**Figure 7:**
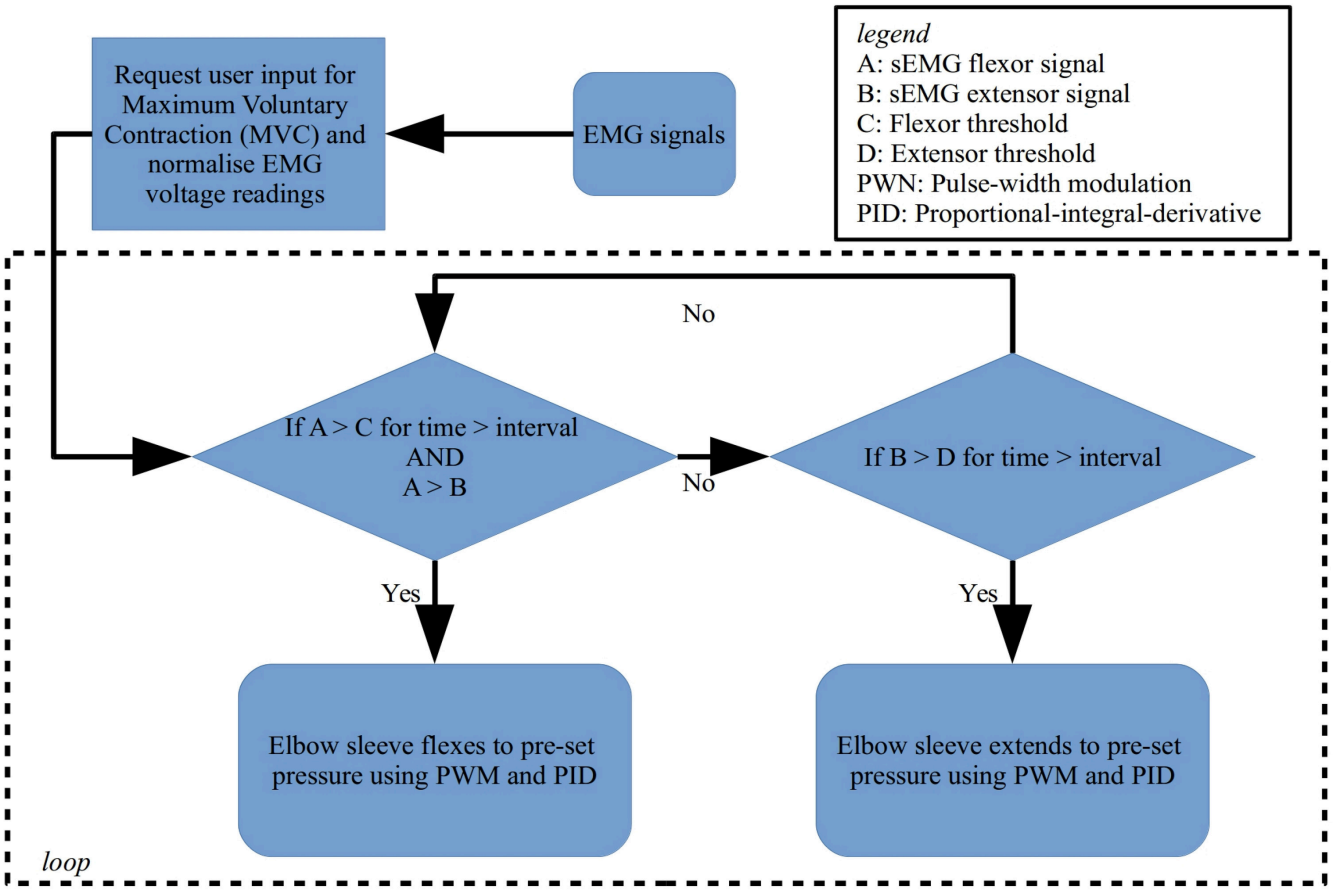
Flowchart of the logic scheme used to detect user intent and control flexion and extension of the soft robotic elbow sleeve.

Conversely, in active intent-based rehabilitation, the detection of user intent is achieved by the surface EMG sensors attached to the biceps and triceps, in which the EMG signals are passed through a logic sequence to allow for flexion or extension of the elbow. The EMG control scheme uses rectified, amplified and then integrated surface EMG signals captured from the biceps and triceps. Notably, the nature of the surface EMG signals captured is highly dependent on their spatial location from the muscle fibers, which is difficult to regulate with high precision. The strength of the EMG signals also typically changes with different subjects as well as different muscles (Halaki and Ginn, 2012). To rectify this, for comparison purposes, both signals from the biceps and triceps are normalized by first requesting maximum voluntary contraction (MVC) effort from the user in an initiation sequence. The MVC value obtained is then used as a reference point against which all muscle signals are compared to during the task. This allows assessment of the level of activity and improves the reproducibility of the data. The signals are subsequently passed through a series of conditions as shown in Figure 7 that cause the activation and deactivation of the actuators, based on a time-over-threshold algorithm. For example, the condition for flexing is met when the processed signal from the bicep crosses a percentage threshold for an interval of 20 ms, while ensuring that the signal from the biceps is stronger than the signal from the triceps. A similar set of conditions exists for extension to occur. This method of control minimizes the occurrence of involuntary muscle contractions that may cause misinterpreted commands.

### 2.5. Range of motion study

Six healthy human subjects (20–25 years old), three males and three females, were enrolled in this study. Written and informed consent were obtained from all the subjects, with approval from the university's Institutional Review Board (NUS-IRB) prior to the experiment. All experimental trials were performed in a gait analysis laboratory.

Each trial comprises of three segments: (1) voluntary active elbow flexion and extension without the elbow sleeve; (2) use of the elbow sleeve in passive rehabilitation mode; and (3) use of the elbow sleeve in active rehabilitation mode.

**Figure 8:**
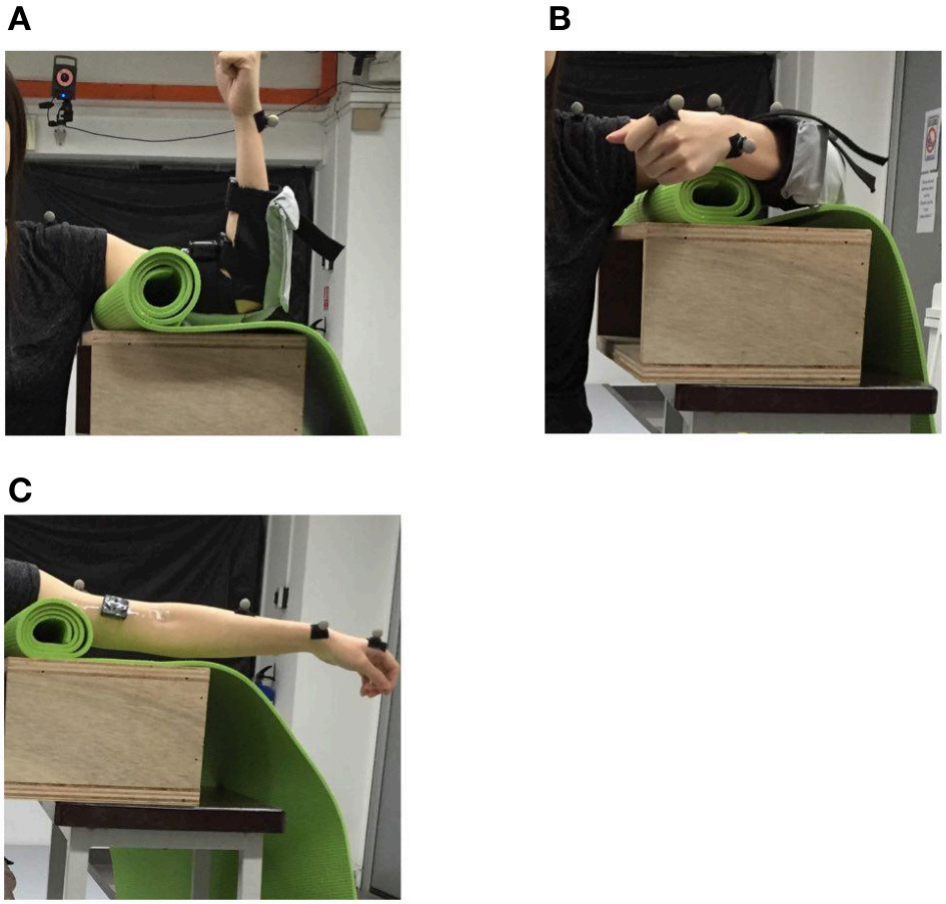
The positions used in the range of motion study. **(A)** Vertical position of the elbow (in flexed configuration). **(B)** Horizontal position of the elbow (in flexed configuration). **(C)** Extended elbow configuration, which serves as the starting point for the trials.

In the first experimental segment, subjects were seated and requested to perform three vertical elbow flexions and extensions in succession, for a total of three trials, followed by three horizontal elbow flexions and extensions in succession for another three trials (Figure 8). All trials were started from an extended elbow configuration of the subjects (Figure 8C). This is representative of activities of daily living such as lifting an object (vertical motion) as well as wiping a table (horizontal motion).

In the second experimental segment, the subjects were then tasked to perform the trials in the first segment, with assistance provided by the soft robotic elbow sleeve in the absence of muscular signal input. After donning the soft robotic elbow sleeve, the passive mode of the elbow sleeve system was engaged, causing the subjects' arms to move in the same sequence of activities as in the first segment.

In the third experimental segment, the control mode of the elbow sleeve was switched to the active rehabilitation mode. Two surface EMG sensors (Myoware, Advancer Technologies, LLC) were mounted on the bicep and triceps muscle groups on the arm without the elbow sleeve. The subjects were then tasked with completing only horizontal elbow flexion using activation signals from the two muscle groups on the free arm to control the state of the elbow sleeve.

**Figure 9:**
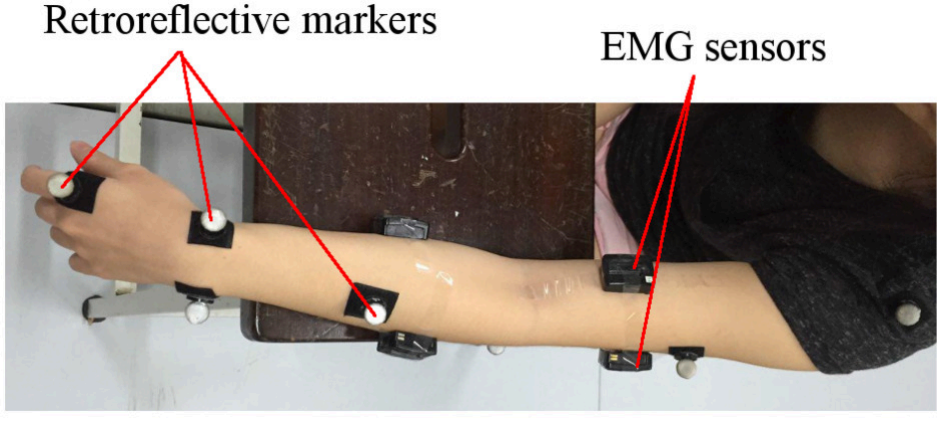
Marker positions for real-time tracking of segment coordinates.

The Vicon motion capture system was used to track the arm positions of the subjects. Retroreflective markers were attached to each subjects' arm at positions shown in Figure 9, and their 3D coordinates were sampled at a frequency of 100 Hz. At each sampling point, the resultant elbow flexion angle, formed between the shoulder-to-elbow segment and the elbow-to-wrist segment (as marked out by the retroreflective markers), was computed and recorded for analysis. As such, a fully extended elbow (no flexion) corresponded to a flexion angle of 0°. The range-of-motion (ROM) thus refers to the maximum angle of elbow flexion attainable by each subject.

For the recording of EMG signals from the muscles, the Delsys Trigno Wireless EMG system was used, with a sampling frequency of 1,000 Hz. To minimize the effects of skin resistance, the area was swabbed with alcohol prior to the placement of the EMG sensors on the biceps and triceps. The inherent variability of the EMG signals necessitates post-processing of the recorded signals. In lieu of this the EMG signals were full-wave rectified, filtered with a second-order band pass Butterworth filter and normalized against the maximum muscle signal output during the first experimental segment.

## 3. Results

### 3.1. Actuator characterization

**Figure 10:**
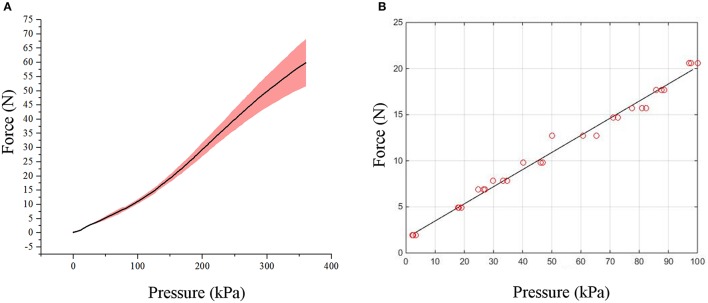
Graphs showing the relationship between force and pressure for the actuators. **(A)** Graph of tip force vs. pressure of flexion actuator. **(B)** Graph of resistive force vs. pressure of extension actuator.

The experimental results of the bending force experiments conducted on the flexion actuators are presented in Figure 10A, showing a reproducible non-linear relationship between input pressure and tip force. The maximum force measured at a pressure of 350 kPa was 67.4N, which is well above the sufficient 20N required for the actuation of the elbow (data acquired from body segment data). A force higher than 20N is necessary to compensate for the forces which are required to bend the actuator itself and ensure that sufficient bending force is supplied to the arm segments.

For the extension actuator, the pressure-force relationship showed a linear trend (Figure 10B). Linear regression was performed on the data to fit a calibration line for the elbow sleeve (*R*^2^ = 0.9951), which allows for an estimation of the forces produced at varying pressure magnitudes. At 80kPa, the average deflective force of 16.5N the actuator produced was capable of enabling full elbow extension.

### 3.2. Control characterization

**Figure 11:**
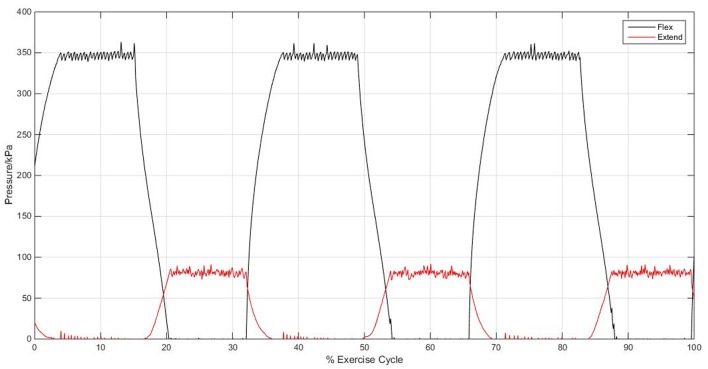
Graph showing the functionality of the PID control of pressure within the actuators.

Figure 11 shows the control of pressure within the actuators for the passive rehabilitation mode of the elbow sleeve. The PID controller system was able to maintain pressure at 350 kPa for the flexion actuator and 80 kPa for the extension actuator with small variations of approximately 10 kPa. This is adequate for generating the necessary flexion and extension for the elbow joint in the range of motion study.

### 3.3. Range of motion study

#### 3.3.1. Efficacy of soft robotic elbow sleeve

To quantify the efficacy of the elbow sleeve in assisting elbow flexion and extension, the elbow joint angles measured in the course of the first and second experimental segments were compared. The first experimental segment involved voluntary active elbow flexion and extension without the elbow sleeve while the second segment involved the use of the elbow sleeve in passive rehabilitation mode, flexing and extending a still elbow.

**Figure 12:**
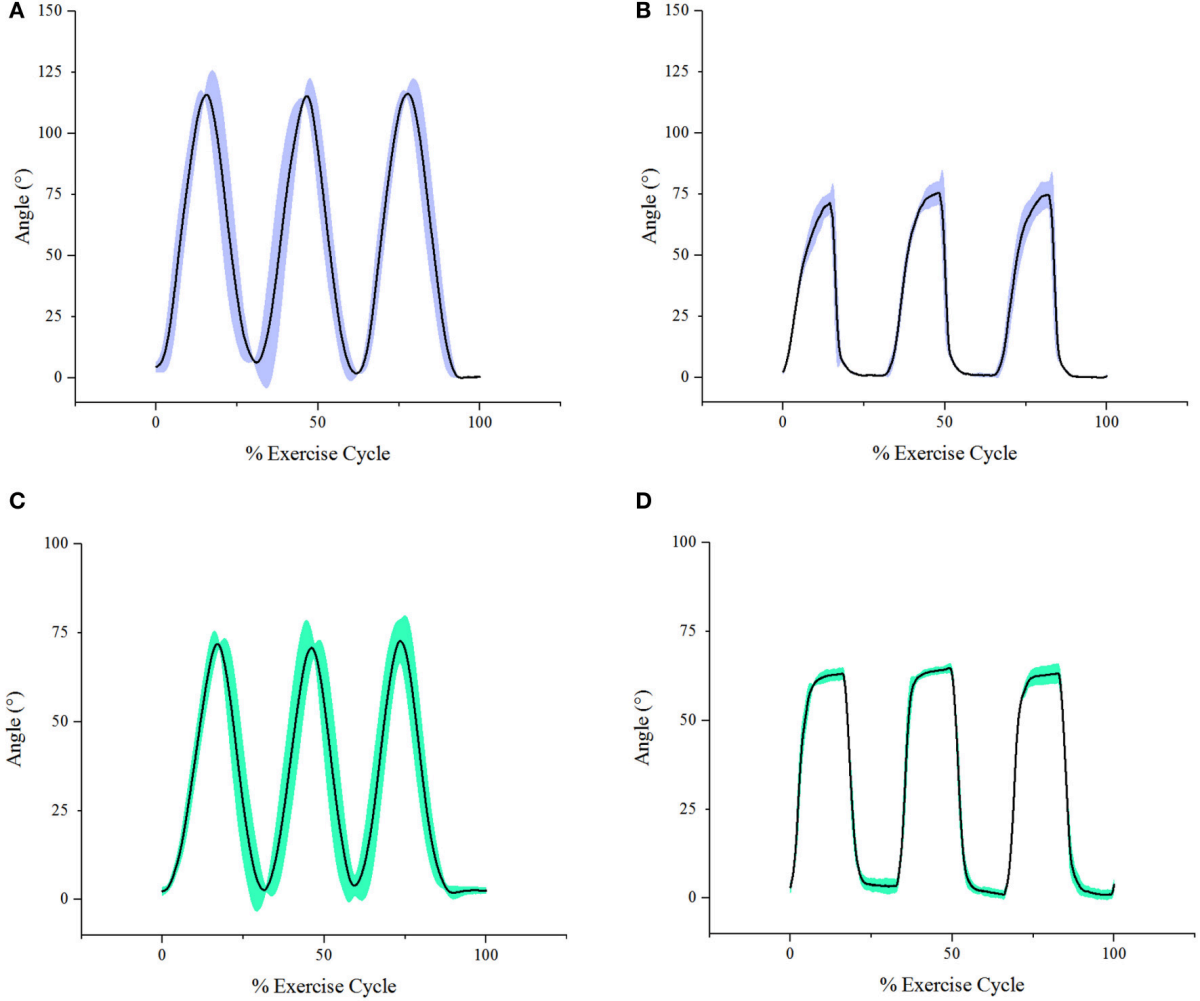
Graphs of angle achieved during exercise cycle (with shaded area depicting standard deviation) of a single subject for each sub-experiment. **(A)** Active vertical sub-experiment. **(B)** Passive vertical sub-experiment. **(C)** Active horizontal sub-experiment. **(D)** Passive horizontal sub-experiment.

In each experimental segment, elbow flexion and extension were performed in two different directions, vertically and horizontally. As a representation, Figure 12A shows a single subject's elbow joint angle from three cycles of active elbow flexion and extension (experimental segment 1), while Figure 12B shows the same subject's elbow joint angle under passive contraction (experimental segment 2). Looking at the results from the different genders, for the female subjects, it was found that the average active maximum ROM was 127.8° while the average passive maximum ROM was 69.3°. This translates to the soft robotic elbow sleeve's capability in achieving 54.2% of the active ROM (with standard deviation of 27.2°). In contrast, for male subjects, it was found that the average active maximum ROM was 132.1° while the average passive maximum ROM was 59.7°. This meant that the robotic sleeve was able to achieve 45.2% of the active ROM (with standard deviation of 23.4°).

For the results in the horizontal direction, as a representation, Figure 12C shows a single subject's elbow joint angle from three cycles of active elbow flexion and extension (experimental segment 1), while Figure 12D shows the same subject's elbow joint angle under passive contraction (experimental segment 2). For the female subjects, it was found that the average active maximum ROM was 118.9° while the average passive maximum ROM was 63.3°. This translates to the soft robotic elbow sleeve's capability in achieving 53.2% of the active ROM (with standard deviation of 15.6°). In contrast, for male subjects, it was found that the average active maximum ROM was 103.3° while the average passive maximum ROM was 56.1°. This meant that the robotic sleeve was able to achieve 54.3% of the active ROM (with standard deviation of 9.7°).

The results for the different genders across the two experimental segments are summarized in Table 1. In all trials, extension of the elbow back to the starting point was achieved.

**Table 1:** Summary of experimental results for segments 1 and 2.

	**Measurement**	**Average (°)**	**Standard deviation (°)**
Female	Active (vertical)	127.8	6.7
	Passive (vertical)	69.3	27.2
	Active (horizontal)	117.9	15.0
	Passive (horizontal)	63.3	15.6
Male	Active (vertical)	132.1	15.05
	Passive (vertical)	59.7	23.4
	Active (horizontal)	103.3	24.1
	Passive (horizontal)	56.1	9.7

#### 3.3.2. Electromyography-driven active control

We reproduce here the results of the third segment of the ROM study, in which the control mode of the elbow sleeve was switched to the active rehabilitation mode, and the subjects were tasked with completing horizontal elbow flexion and extension cycles using only activation signals from the two muscle groups on the opposite arm (non-sleeved arm) to control the state of the elbow sleeve.

**Figure 13:**
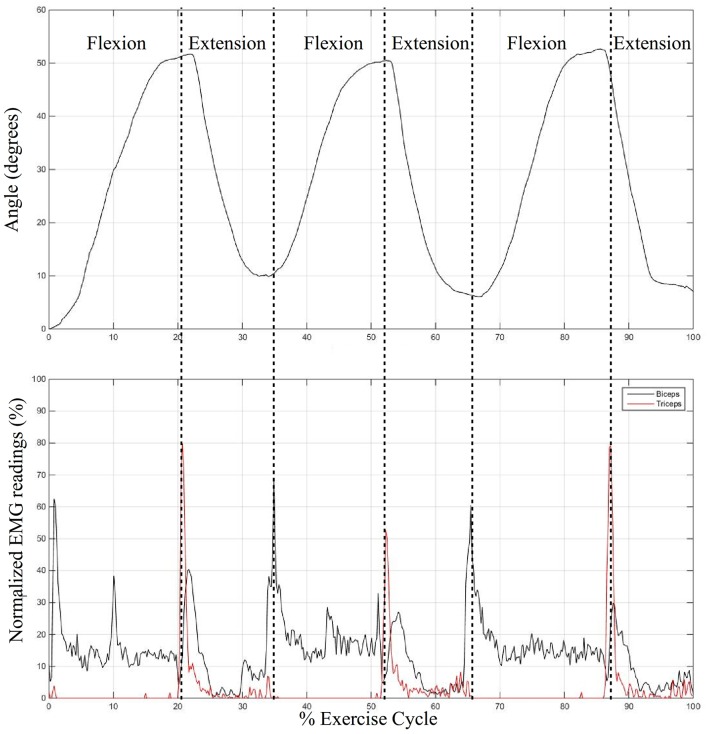
Graph showing the angle of the elbow over the exercise cycle corresponding to the captured surface EMG signals over time from the biceps and triceps, in cyclic flexion and extension.

All the subjects were successful in controlling the state of the elbow sleeve with muscle effort from the target muscle groups detected by the surface EMG sensors. As a representation, Figure 13 shows the processed EMG data from the biceps and triceps of a single subject normalized to the threshold of 50%, together with the change in elbow joint angle upon EMG activation by the user. It was observed that there is a tendency for co-contraction during extension (as observed by high EMG signals from both muscles). This was possibly due to the action of joint stabilization by both muscle groups, which did not affect the functionality of the elbow sleeve, for the algorithm continues to function so long as the threshold is met by the intended muscle.

## 4. Discussion

In this paper, we presented the design and testing of a soft robotic elbow sleeve that could be used for passive and intent-based neuro-muscular training. The novelty of this design comes from the use of lightweight fiber-reinforced and fabric-based soft actuators for elbow moving, and this device represents the first of its kind in integrating both flexion and extension capabilities. The elbow sleeve's capability in enabling full extension is particularly useful for stroke patients. In studies assessing the motor impairment and activity limitations of stroke patients, it was found that spasticity, which is a velocity-dependent hyper-excitability of muscles to stretch, is a common complication encountered, at an estimate of 60% of post-stroke patients (Watkins et al., 2002; Sommerfeld et al., 2004). This is manifested in the form of a slightly flexed elbow with the forearm across the abdomen. Another common problem that arises in stroke is that of muscle contracture, which also leads to similar complications as spasticity. Typical physiotherapy treatments for such ailments include slow progressive elbow extensions, which has seen reasonable success for ROM reestablishment (Bonutti et al., 1994). As such, this means of therapy can be potentially fulfilled by our elbow sleeve due to the inclusion of extension actuators.

An important design constraint—that of ensuring that extension actuators do not hinder the flexion actuators—determined that each of the actuators has to be manufactured differently. In this regard, the flexion actuator is a fiber-reinforced silicone actuator coupled with nylon fabric to influence its bending behavior, whereas the extension actuator is a inflatable fabric actuator which provides resistive forces through a beam bending model. Both actuators show differences in their pressure-force relationships, with the flexion actuator exhibiting a nonlinear sigmoid-shaped curve, and the extension actuator showing a linear relationship. The non-linearity of the flexion actuator is largely due to the anisotropy that is incorporated within the flexion through fiber and fabric reinforcements, while the extension actuator displayed a linear pressure-force relationship in the absence of modifying reinforcements to the material.

In examining the control system of the elbow sleeve, while it is successful in generating and maintaining the necessary flexion and extension for the elbow joint in the ROM study, there remains an undesirable frequency switching which led to a slight oscillatory movement of the elbow sleeve during actuation. To overcome this, a dead-zone can possibly be introduced at the valve controller, creating boundaries around the pressure readings that allow the valves to remain at their current states until the change in pressure is sufficiently large, which is a suggested method by Guo et. al on modifications to improve the PID controller (Guo et al., 2002).

Preliminary studies were carried out on healthy subjects to quantify the basic functionality of the elbow sleeve. From our results across all the experiments, it was observed that while the extension actuators were able to ensure full extension of the elbow, the flexion actuators had difficulties in enabling full flexion of the elbow, achieving an average of approximately 50% of the full ROM across all subjects. As such, there is room for improvement with respect to the ROM achievable by the device. To improve the ROM, it is recommended that finite element modeling of the fiber-fabric-reinforced actuators be conducted to determine the maximum possible force output of these actuators. Once the full potential of the actuators is quantified in these computational models, feedback control loops may then be implemented to control the extent of actuation by monitoring the angle that the elbow is flexed to. Additionally, alternative materials for actuator fabrication, or an increase in the number of flexion actuators on the device may also be considered.

In comparing the results gathered from each gender, it was observed that the average percentage ROM achieved from using the elbow sleeve was lower for the male subjects, which was expected due to male subjects having a generally higher segment mass as compared to female subjects. On a larger front, this analysis also suggests that there is a need to tailor the mechanical properties of the actuators to fit each individual's requirements.

In comparing the results gathered from elbow motion in the vertical and horizontal directions, it was observed that the variance in vertical motion is lower as compared to that in the horizontal direction of motion. This is possible due to the decreased effects of gravity acting in the horizontal direction as compared to the vertical direction, thereby making it easier to flex the elbow horizontally as compared to vertically. This set of results is useful in further optimizing the control system toward sensing and providing different bending forces suited for the mechanical task at hand.

The EMG mode of control was successfully implemented during the preliminary user studies. This indicates the functionality of the elbow sleeve as a means of providing active-assisted movements. Going forwards, by placing the surface EMG electrodes on the non-supported arm, bimanual practice may be enabled. The significance of this in stroke recovery is that the consensual operation of the non-affected upper limb may cause the stimulation of ipsilateral corticospinal projections to the paretic muscles, which is of relevance to hemiplegic recovery (Hesse et al., 2003). The logic scheme used for the EMG control is primarily based on an on-off algorithm, and a possible improvement to the system would be to integrate angular control into the device by modifying the algorithm. If bimanual control is maintained, two flex sensors calibrated to obtain angle of bending may be placed onto the back of the elbow. Through comparison of the angle bent by both elbows, a third PID regulator may be introduced to identify the error and adjust the supported elbow, using the non-supported elbow as the set-point angle. Another possible solution would be to record signals from more muscle groups. The array of signals and their amplitudes from these muscle groups would then provide a response pattern as type of fingerprint that may be used to identify different points of elbow actuation. These signals may be classified using either a fuzzy approach or an artificial neural network approach (Chan et al., 2000). In this manner, the surface EMG sensors may be placed on the supported arm to detect post-stroke using residual muscle signals.

Finally, the testing of the system was done with six healthy subjects. While this is sufficient for the purpose of demonstrating the functionality of the system in enabling robot-assisted flexion and extension of the elbow with and without intent detection, clinically-relevant conclusions may not be reached as the system has to be tested on individuals with neurological impairments. A limitation of testing healthy subjects is such that, healthy subjects, in contrast with individuals with neurological impairments, have a tendency to activate their agonist muscles during assistive movement. In this study, we found one subject to exhibit biceps activation during passive mode rehabilitation. Moving forwards, the next step to this study would be to conduct clinical trials on stroke patients, who are the intended end-users of the elbow sleeve. These patients would present differing physical conditions (such as their EMG signal amplitudes) which would better elucidate the efficacy of the elbow sleeve, and the improvements that needed to be made.

## Author contributions

TK and NC collected, processed and analyzed the data, drafted the manuscript and contributed equally to the work. TK, NC, HY, and CY designed the study. HY contributed to the interpretation of findings and drafted the manuscript. CY oversaw its coordination and helped to draft the manuscript. All authors read, edited and approved the final manuscript.

### Conflict of interest statement

The authors declare that the research was conducted in the absence of any commercial or financial relationships that could be construed as a potential conflict of interest.
